# Non interventional drug studies in oncology: Why we need them?

**DOI:** 10.4103/2229-3485.71770

**Published:** 2010

**Authors:** Divya Mishra, Jesal Vora

**Affiliations:** *Division of Medical and Research, Pfizer Limited Patel Estate, Off S.V. Road, Jogeshwari (West), Mumbai - 400 102, Maharashtra, India*

**Keywords:** Good clinical practice, non interventional studies, standard operating procedures, study plan

## Abstract

Oncology is a highly researched therapeutic area with an ever expanding armamentarium of drugs entering the market. It is unique in how the heterogeneity of tumor, patient and treatment factors is critical in determining outcomes of interventions. When it comes to decision making in the clinic, the practicing physician often seeks answers in populations with obvious deviations from the ideal selected populations included in the pivotal phase III randomized controlled trials (RCTs). While the randomized nature of the RCT ensures its high internal validity by removing bias, their ‘controlled’ nature casts a doubt on their generalizability to the real world population. It is for this reason that trials done in a naturalistic setting post the marketing authorization of a drug are increasingly required. This article discusses the importance of non interventional drug studies in oncology as an important tool in testing the external validity of controlled trial results and its value in generation of new hypothesis. It also discusses the limitations of such studies while outlining the steps in their effective conduct.

## PURPOSE/AIM

To discuss the benefits and possible challenges of non interventional drug study designs and their conduct in oncology clinical arena.

## BACKGROUND

Cancer is unique in how the heterogeneity of tumor, patient and treatment factors is critical in determining outcomes of interventions. Cancer clinical trials in various stages of drug development are increasingly becoming multicentric and multinational and are being conducted across different geographies with diverse patient demographics and differing incidence, prevalence, clinical course of the disease in question and co morbidities. This is a welcome development for the policy makers and practitioners as the data applicability and extrapolation challenges between different settings of patients are getting minimized.

However, the limitations associated with restrictive eligibility criteria or incompleteness of AE reporting or adequate description of other determinants of external validity[1] still remain as an integral limitation of RCTs. Reporting of adverse effects of treatment in RCTs and systematic reviews is often poor. In a review of 192 pharmaceutical trials, less then a third had adequate reporting of adverse clinical events or laboratory toxicological findings.[2] Treatment discontinuation rates provide some guidance to tolerability but pharmaceutical trials often use eligibility criteria and run-in periods to exclude patients who might be prone to adverse effects. Rates of discontinuation of treatment are therefore greater in clinical practice.[3,4] Publication bias and inadequate reporting of adverse events in RCTs supported by the pharmaceutical industry is a longstanding and unresolved difficulty.[5,6] Coupled with these, there is a persisting concern among clinicians that external validity is often poor, particularly for some pharmaceutical industry trials, a perception that has led to under use of treatments that are effective.

The point of generalizability is not to address whether there is similarity between the groups compared within a trial, where an imbalance or lack of “internal validity” would be a form of bias, but rather whether the groups studied resemble the population to whom the trial results might be applied, thus making these results “externally valid.” The importance of considering the generalizability of a trial’s results is highlighted by inclusion of this topic as a quality indicator of RCT reporting within the CONSORT statement.[7]

In addition, in oncology clinical trials, the benefits of interventions over existing standard comparators are becoming increasingly small and so the issues of patient’s preference coupled with the physician’s own experiences are assuming a greater role in the oncologists’ prescription decisions. Patient’s preferences have recently been highlighted as a potential threat to the validity of RCTs. Preferences are generally expected to influence decisions about randomization, but not determine them. In one RCT, 82% of patients willing to enter a comprehensive cohort study agreed to be randomized, but 80% reported having a preference after randomization.[8] Distinguishing between preferences and decisions is important: When patients make randomization decisions that conflict with their preferences, those preferences could still influence outcomes, and thus threaten the validity of the trial. For example, some women with early breast cancer have a strong preference for lumpectomy, whereas others are far happier in the belief that all the cancer has been removed by a mastectomy. However, only women who did not have a strong preference for a particular treatment could be recruited into the relevant RCTs, and as few as 10% agreed to have their treatment chosen at random.[9] If RCTs show a major advantage for one treatment, then external validity is not a problem. Difficulties arise when one treatment is only moderately more effective but the patient has a strong personal preference for the less effective option. Would the results of the breast surgery RCTs, particularly in relation to psychological wellbeing, have been the same if such patients had been randomized?

The importance of physician’s experience and observation in the real world treatment of his patients cannot be undermined. It is for this reason that trials done in a naturalistic setting post the marketing authorization of a drug are increasingly required.

## DEFINITION

Non interventional drug studies are trials conducted to assess safety, tolerability and effectiveness of marketed medicines in clinical practice, i.e. in a naturalistic setting where the choice of therapy is consistent with approved marketing authorization and in line with the current standard of practice. The patient selection and the diagnostic or monitoring procedures are those applied per the usual treatment paradigm of the treating physician and not per protocol.[10]

These studies are, therefore, more representative of the general population than RCTs and help gain insights into baseline prognostic categorization of patients (where applicable), dosing strategies and safety aspects including adverse event management practices and treatment effectiveness (rather than efficacy) [Table 1] and may even bring to the fore, new observations for hypothesis testing.

**Table 1:** Comparison of efficacy and effectiveness trial

Study characteristics	Efficacy trial	Effectiveness trial
Research question	Will the intervention work under ideal conditions?	Will the intervention result in more good than harm under usual practice conditions?
Setting	Restricted to specialized centers	Open to all institutions
Patient selection	Selected, well-defined patients	A wide range of patients selected using broad eligibility criteria
Study design	Smaller RCT using parallel group or factorial or other approaches (crossover design)	Large multicenter RCTs using parallel groups or factorial cluster
Baseline assessment	Elaborate and detailed	Simple and clinician friendly
Study intervention	Tightly protocolized using optimal therapy under optimal conditions	Implemented in usual clinical Practice; limited study protocol if any
Co-interventions	Tightly controlled protocol for many aspects of care	All therapy based on local clinical practice/experience/minimal control
Compliance	Compliance essential	Non compliance expected and considered in sample size / analysis
Analysis	May be done by treatment received where non compliant patients may be removed	Always intention to treat where all patients are included
Data management		
i) Data collection	Elaborate	Minimal and simple
ii) Data monitoring	Detailed and rigorous	Minimal
Study management	Significant interventions and support from research staff	Minimal support and interventions from research team

*Data monitoring refers to the review of source documents and adjudication/verification of outcomes^11^

## STEPS IN STUDY CONDUCT[12]

The sponsoring institutions or pharmaceutical companies can partner with contract research organizations (CRO) and site management organizations (SMO) to enable collection and monitoring of such data so as to ensure high quality data is made available for the practicing physicians. As a broad guideline, the following steps should be followed for successful execution. While the practicing physician may also conduct non interventional drug studies, the use of terminology in the steps outlined below mainly pertains to pharmaceutical company sponsored studies.

### Protocol finalization

The product physician authorizes the protocol (non interventional study plan) and works with relevant approvers for the finalization of the same. It is important that product physician works with statistician to arrive at appropriate sample size estimation. This will help to achieve the objective of the study.

### Budgetary allocation

Budget will have to be finalized for the conduct of the study with approvals from the business unit and other relevant approvers. The grants to the investigators should not provide undue influence to prescribe the product.

### Informed consent and data privacy

Where personal information on study subjects in a non-interventional study is to be used, processed, transferred or disclosed, an appropriate data privacy statement is required and the appropriate consent must be obtained from the study subjects by the study site. The data privacy statement must be compliant with the legal and regulatory requirements in the country where the data is collected, and the local legal department should be involved in drafting a suitable statement.

### Case report form finalization and statistical analysis plan generation

The product physician works with data management to finalize the Case report form finalization (CRF) and statistical analysis plan (SAP). To be effective in startup time, the CRF and SAP can be generated parallel during the protocol approval phase. This is prepared based upon the protocol and standard operating procedures(SOPs). A standardized CRF serves as a tool for systematic collection of data, thus facilitating comparative analysis. This contributes to better quality data and will also answer all questions for such type of studies and also meet the objective of the study. SAP should be finalized including a description how to handle missing or implausible data (e.g. indicate imputation strategies). This strategy ensures the transparency and consistency of the analysis strategy.

### Study monitoring plan

Study monitoring plan should be prepared for such type of studies. This will ensure the sponsor that the trials are adequately monitored. The sponsor should determine the appropriate extent and nature of monitoring. The determination of the extent and nature of monitoring should be based on considerations such as the objective, purpose, design, complexity, blinding, size, and endpoints of the trial. In general, there is a need for on-site monitoring, before, during, and after the trial; however, in exceptional circumstances, the sponsor may determine that central monitoring in conjunction with procedures such as investigators’ training and meetings, and extensive written guidance can assure appropriate conduct of the trial in accordance with good clinical practice (GCP). Statistically controlled sampling may be an acceptable method for selecting the data to be verified.

### Data management plan

This should include a minimum of data flow plan; case report form completion guidelines; data entry methods and guidelines; data validation document and data handling conventions. The database has to be prepared as per the CRF. A data clarification form is required for handling data queries. Also medical terms need to be coded with appropriate medical dictionaries.

### Investigator selection (feasibility conduct and budgets finalization)

Should work with local commercial/ marketing medical colleagues for appropriate site selection making sure that the site will have the appropriate patients. For instance, there is no point in going to sites where patients cannot afford to take medicine or do not have the appropriate medical insurance to cover its costs. There is no real financial motive for the physicians to be involved in the study as per patient costs for these types of studies are generally very low. They need to have an understanding of how they are contributing to research and publications.

Essential documents collection (curriculum vitae [CV] of investigator etc.): This is not the same as required for the interventional studies (Phase I, II and III). Need to comply with the sponsors’ SOPs and local country regulations if any.

### Local regulatory approvals if applicable

This may not be applicable in most countries. Some sponsors have a post marketing authorization commitment to regulatory agency for conducting such studies.

### Institutional review board/independent ethics committee approvals

An opinion from the Institutional review board (IRB)/ independent ethics committee (IEC) is recommended. Complete transparency has to be ensured.

### Site initiation

All the participants from sites including the CRO/SMO have to be selected carefully and trained on the various project specific aspects. This should cover the objective of the study, study plan and case report form. This would also be the ideal situation to train the sites on GCP.

### Patient enrollment

The amount of patients enrolled per site/investigator should be limited to avoid few sites or one site dominates the results and therefore compromises the representativeness. Even distribution of the sites across the region should be ensured. No patient recruitment strategies to be implemented by the sponsoring companies for patient enrollment in such type of studies.

### Data monitoring and CRFs collection

The completed CRFs received are checked for completeness. Trained SMO can be appointed to ensure complete data entry in CRFs. 100% source data verification is not mandatory for such studies. This will depend on the SOPs of the sponsoring company and local country regulations. The main objective parameter, primary variable and safety parameters have to be monitored. Discrepancies can be resolved by sending queries to the site. CRF collection should be done on a regular basis to ensure timely receipt of the same and for data analysis and query resolution.

### Data analysis

The verification and validation of the data is done in agreement and with the help of the investigator. Any data which is incomplete, ambiguous and not readable should be queried to the investigator by the data management. Cross checking of all variables and data review meetings before the database lock should be done with all parties involved to discuss data and queries if any. The analysis should be standardized through standard tables and figures which will help in use of standard analysis program. This will not only help in cost and time savings but also help in having comparable results in between different studies.

### Report writing

The final report which is developed after the analysis should reflect clearly all activities and methods within the scope of the study. All the study results are presented and information is hidden. There is complete objectivity in interpretation of results. Presentation of the used study design, the predefined hypothesis, the complete description of statistical methods, the details of the data management activities and the discussion of possible sources for bias of the results should be included in the report.

### Publication of results

The study has to be registered in Clinical Trials.gov and the results need to be posted on ClinicalStudyResults.org. In case publication is not possible, atleast a summary of results should be published in the public registry.

Most of the steps outlined above are also relevant in the conduct of an RCT. However, there are differences in terms of additional resource intensive activities required for an RCT. These include the following:

- Randomization process: This needs to be clearly outlined in the protocol and necessary arrangements for implementation (e.g. interactive voice response system [IVRS]) need to be ensured.
- Drug supply: Sponsor usually supplies drugs for the treatment arms and ensures compliance to local country regulatory and company’s SOPs.
- Protocol procedures: Strict adherence to laid out criteria in the protocol has to be ensured.
- Regulatory approval: This is a mandatory requirement besides ethics committee approvals in most countries.
- Source documents: 100% source data verification is required.

## DISCUSSION

Drug research and surveillance after authorization has become more and more important for several reasons. The reasons could be ranging from post marketing authorization commitment to the country regulatory authority or even a simple observation of drug effects in the population of interest.

Different types of Phase IV studies may be conducted for addressing different questions, and not always pertaining to the direct effects of the use of a drug. These include the following:

- Post marketing surveillance studies: Subsequent to approval and marketing of the product, close monitoring for their clinical safety is undertaken. This needs to be submitted in the form of periodic safety update reports (PSURs) to local regulatory agency.
- Drug utilization studies: Such studies describe how a drug is marketed, prescribed, and used in a population, and how these factors influence outcomes, including clinical, social, and economic outcomes.
- Registry: Ongoing and supporting data over time on well defined outcomes of interest is collected on patients with certain shared characteristics (e.g., particular disease, exposure, or risk factor) for analysis and reporting.
- Case controlled studies: Rare suspected side effects can be evaluated and reported through such studies.
- Non interventional studies: These can be drug studies and non drug studies.

Non interventional drug studies investigate various aspects of drug use including effectiveness, safety and tolerability under real life conditions. Unlike RCTs, NIS is less resource and capital intensive. Therefore, conduct of such kind of research should be a priority on a very high scientific and methodological basis. Key elements include identifying a scientific need for drug related data generation; developing a study plan/protocol containing the scientific objective; a sample size justification; a description of the various activities to be performed during the conduct of the study; a description of planned analyses and the publishing of summary of results timely after completion of the study. The quality and the study conduct can be improved by selecting the most appropriate investigator; assigning the relevant budgets; using standardized case report form (CRF) and statistical analysis plan (SAP). The development of own standard operating procedures (SOP) describing the processes during planning, conduct and evaluation of a non-interventional study as well as the quality management and the regular training of all involved people is also highly recommended. All accompanying measures to improve or to keep the quality of the non interventional drug studies should not violate the concept of non-intervention.

The reason for conducting NIS is to present an actual original picture of the current routine medical practice. It is not intended to encourage healthcare professionals or customers to recommend, prescribe, purchase, supply, sell or administer a medicinal product. Differentiating between intervention and routine medical practice would be challenging. Currently whether the assignment of written informed consent form and patient questionnaires/diaries violate the nature of the non intervention is not yet clear. The informed consent would confirm compliance according to the policy of privacy of personal information. But this will always finally depend on the institutional review board (IRB)/independent ethics committee (IEC). NIS is not a promotional exercise. Sales and marketing colleagues may be involved only in administrative capacity subject to local laws, regulations and permissions. It can also provide epidemiological information about a particular disease, or even identify an unfulfilled medical need.

### Merits

- Simplistic study design mandates no deviation from current medical practice.
- Helps to derive the best generalizable evidence about the effects of or the reactions to a drug.
- Serves as a common platform for practitioners to share best practices and real life experiences through their common endorsed protocol.
- Encourages orientation to GCP and capability development in clinical research for budding investigators.

### Demerits

- Lack of internal validity due to unselected populations and possibilities of bias.
- Tight timelines and an empirical approach to their estimations.
- Low budgets for these studies and lack of provision of the drug as part of the study designs are some of the major challenges in meeting meaningful recruitment targets in the defined timeframe.

## CONCLUSION

Numerous known and unknown variables may impact the applicability of the results from randomized controlled trials (RCPs) to the real world scenarios. This effect may be even more pronounced in oncology where the heterogeneity in patient and disease characteristics is well known coupled with reducing differences in the risk-benefit ratios among newer drugs. In addition, a number of factors influencing outcomes may remain unidentified and unrecognized in the restrictive design of a controlled trial. Non interventional drug studies are therefore vital.
